# Giant stress response of terahertz magnons in a spin-orbit Mott insulator

**DOI:** 10.1038/s41467-022-34375-6

**Published:** 2022-11-05

**Authors:** Hun-Ho Kim, Kentaro Ueda, Suguru Nakata, Peter Wochner, Andrew Mackenzie, Clifford Hicks, Giniyat Khaliullin, Huimei Liu, Bernhard Keimer, Matteo Minola

**Affiliations:** 1 Max Planck Institute for Solid State Research, Heisenbergstraße 1, D-70569 Stuttgart, Germany; 2 Department of Applied Physics, The University of Tokyo, Bunkyo-ku, Tokyo, 113-8656 Japan; 3 Max Planck Institute for Chemical Physics of Solids, Nöthnitzer Straße 40, D-01187 Dresden, Germany; 4 SUPA, School of Physics and Astronomy, University of St. Andrews, St. Andrews, KY16 9SS United Kingdom; 5 School of Physics and Astronomy, University of Birmingham, Birmingham, B15 2TT United Kingdom; 6 Leibniz Institute for Solid State and Materials Research Dresden IFW, Helmholtzstraße 20, D-01069 Dresden, Germany

**Keywords:** Magnetic properties and materials, Magnetic properties and materials

## Abstract

Magnonic devices operating at terahertz frequencies offer intriguing prospects for high-speed electronics with minimal energy dissipation However, guiding and manipulating terahertz magnons via external parameters present formidable challenges. Here we report the results of magnetic Raman scattering experiments on the antiferromagnetic spin-orbit Mott insulator Sr_2_IrO_4_ under uniaxial stress. We find that the energies of zone-center magnons are extremely stress sensitive: lattice strain of 0.1% increases the magnon energy by 40%. The magnon response is symmetric with respect to the sign of the applied stress (tensile or compressive), but depends strongly on its direction in the IrO_2_ planes. A theory based on coupling of the spin-orbit-entangled iridium magnetic moments to lattice distortions provides a quantitative explanation of the Raman data and a comprehensive framework for the description of magnon-lattice interactions in magnets with strong spin-orbit coupling. The possibility to efficiently manipulate the propagation of terahertz magnons via external stress opens up multifold design options for reconfigurable magnonic devices.

## Abstract (web summary)

There has been significant interest in using spin-waves or magnons for information processing, due to their low energy dissipation and short wavelength at terahertz frequencies, however, manipulating magnons can be challenging. Here, Kim et al show that magnons in Sr2IrO4 are extremely strain sensitive, with small applied strains leading to large variation in the magnon energy.

## Introduction

In recent years, 4*d* and 5*d* transition metal oxides have been extensively investigated in the search for novel electronic phases ^1,2^. In these compounds, the spin–orbit coupling (SOC) is comparable to other relevant interactions, such as the exchange and Jahn-Teller couplings, and therefore not treatable as a small perturbation as it is in 3*d*-electron compounds. Instead, spin–orbit-entangled states comprising various *d*-orbitals with a different spin and orbital quantum numbers are formed. These states are represented by an effective (“pseudo”) spin $\widetilde{J}$^3^. The pseudospins have a large orbital contribution and thus generate a nontrivial structure of the orbital interactions, which are bond-directional and highly susceptible to crystal field environments^4^. This leads to the realization of various exotic magnetic ground states, such as the Kitaev spin-liquid on the honeycomb lattice^5–9^ and the all-in-all-out magnetic structure on the pyrochlore lattice^10–12^, with corresponding collective excitations in the terahertz range. A major frontier of current research seeks to understand and control the confluence of spin–orbit, spin-lattice, and exchange interactions that determines the nature of these excitations, and to harness them for device applications.

The Mott-insulating square-lattice iridate Sr_2_IrO_4_ has emerged as a model system for spin–orbit-entangled magnetism and has drawn broad attention as a rare example of the quasi-two-dimensional Heisenberg antiferromagnet, a concept that is of key importance for the cuprate high-*T*_*c*_ superconductors^13^. In this compound, Ir^4+^ ions with ${t}_{2g}^{5}$ configuration possess spin *S* = 1/2 and an effective orbital moment $\widetilde{L}=1$, which are coupled by a large SOC (*ξ* ≃ 0.4 eV), forming a pseudospin $\widetilde{J}=1/2$^14^. Below *T*_N_ = 240 K, Sr_2_IrO_4_ shows canted in-plane antiferromagnetic order. Resonant inelastic x-ray scattering experiments^15^ revealed magnon excitations with energies up to 50 THz, comparable to those in the cuprates.

Whereas the overall energy scale in Sr_2_IrO_4_ is dictated by the isotropic Heisenberg coupling, higher-order interactions are crucial for understanding its low-energy magnetic properties. For example, Dzyaloshinskii–Moriya and XY-type exchange anisotropy terms^6^ are instrumental to describe the weak ferromagnetism and magnetic critical scattering data^16^. However, these interactions do not uniquely determine the staggered moment direction in a crystal. Experimentally, the pseudospins are oriented along the [1 0 0] axis, i.e., 45^∘^ away from the Ir-O-Ir bond direction^14,17^. Raman^18,19^ and inelastic neutron scattering experiments^20^ detected a magnon gap of ~ 0.5 THz associated with the magnetic anisotropy within the *xy*-plane. The nature of the interactions breaking the XY-symmetry in Sr_2_IrO_4_ remains unresolved to-date. In principle, pseudodipolar-type couplings^6^ can open a magnon gap via an order-by-disorder mechanism as in the cuprates^21,22^, but this would align the moments parallel to the Ir-O-Ir bond direction, inconsistent with the experiment.

There have been several theoretical attempts to clarify the origin of the in-plane anisotropy in Sr_2_IrO_4_. Katukuri et al.^23^ suggested that the anisotropic interlayer couplings might be relevant, whereas ref. 24 proposed a mechanism involving orbital-lattice coupling. Via spin–orbit entanglement, this coupling is transformed into a pseudospin-lattice interaction, which breaks the tetragonal symmetry and generates an in-plane anisotropy. A small orthorhombic distortion below *T*_N_ was predicted based on this model. On the experimental front, various manifestations of the pseudospin-lattice interaction in iridates have recently been reported^25–27^. Decisive experimental evidence that establishes a clean, solid connection between the theory and experiments is, however, still lacking.

Here we provide direct evidence that pseudospin-lattice coupling is responsible for the in-plane magnetic anisotropy in Sr_2_IrO_4_, by tuning the magnon gap using in-situ uniaxial strain. Raman spectroscopy is particularly suitable for this purpose, thanks to its capability to detect subtle energy shifts with ~1 GHz energy resolution, combined with the experimental flexibility to combine spectroscopic measurements with uniaxial strain techniques at cryogenic temperatures. We performed Raman scattering experiments on single crystals of Sr_2_IrO_4_ under the uniaxial strain along different crystallographic directions. We found that the magnon gap is remarkably sensitive to small levels of strain. The effect is highly anisotropic and it is symmetric with respect to the sign of the strain, indicating an intimate connection between the in-plane anisotropy and the orthorhombicity. We present a theory that describes our experimental data in quantitative detail and evaluates the intrinsic orthorhombic distortion in Sr_2_IrO_4_ induced by pseudospin-lattice coupling.

## Results

**Fig. 1:**
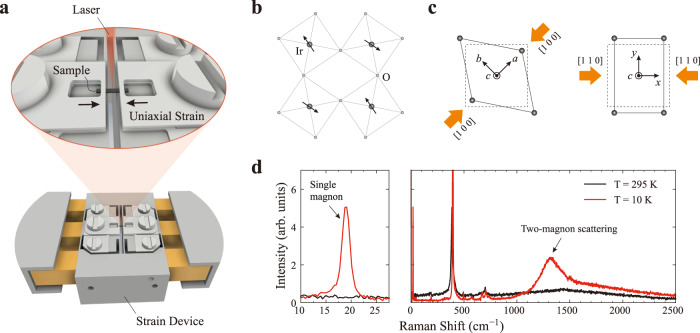
Measurement setup for uniaxial strain experiments. **a** Schematic picture of the experimental geometry with the strain device. **b** Sketch of the crystal and magnetic structure of an IrO_2_ plane. Black (gray) circles represent Ir (O) atoms. Black arrows represent pseudospins. **c** Orthorhombic deformations of *xy* type (left, strain along the [1 0 0] direction) and *x*^2^ − *y*^2^ type (right, strain along the [1 1 0] direction). Solid (dashed) lines represent the strained (undistorted) lattice. Orange arrows show the applied stress directions. **d** Raman spectra of *B*_2g_ symmetry measured below and above *T*_N_. The left panel shows the low-energy range, where a single-magnon peak is observed below *T*_N_.

For strain control, we employed a modified version of the piezoelectric-based strain device successfully utilized in several studies of correlated-electron systems^28–33^. Figure 1a shows a schematic picture of the experimental geometry (see Methods for details). We chose two different geometries with uniaxial strain along [1 0 0] and [1 1 0], as shown in Fig. 1c. The strain levels were first estimated using a built-in displacement sensor during the Raman measurements, and later recalibrated by comparison to intrinsic observables measured at each displacement value. To this end, we carried out separate measurements of the energies of selected phonons and of the positions of selected in-plane Bragg reflection by Raman and x-ray scattering, respectively (See Supplementary Note 1 for the strain calibration procedure). Throughout this article, we use the convention of Fig. 1c to represent the crystallographic directions and the polarization geometry.

**Fig. 2:**
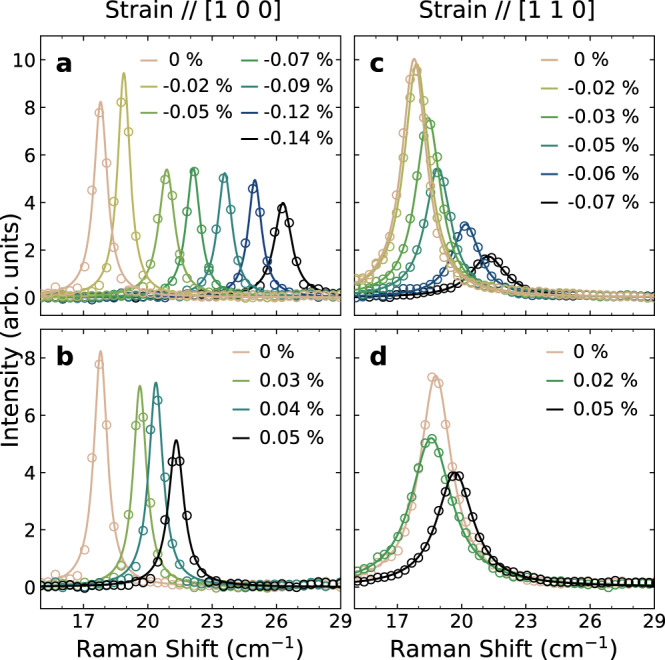
Raman spectra of single-magnon excitations under uniaxial strain. Background-subtracted low-energy Raman spectra were measured under **a** compressive and **b** tensile strain along the [1 0 0] direction and under **c** compressive and **d** tensile strain along the [1 1 0] direction. Solid lines are the results of Lorentzian fits to the data.

Figure 1d shows magnetic Raman spectra with *B*_2g_ symmetry. Well below *T*_N_ ~240 K, where the pseudospins align antiferromagnetically as illustrated in Fig. 1b, we observe a sharp single-magnon peak at 18 cm^−1^ (0.54 THz) and a broad two-magnon scattering signal centered at 1300 cm^−1^ (39 THz), which had already been identified in the literature^18,19,25,34^. Figure 2 displays Raman spectra of single-magnon excitations at the zone center under uniaxial strain applied along the [1 0 0] and [1 1 0] axes. Under compressive strain along [1 0 0], the single-magnon energy increases markedly and monotonically, as shown in Fig. 2a. The peak is still clearly visible at *ε*_[100]_ = −0.14%, although it broadens slightly and loses some of its intensity with increasing strain. Similar results are found under the tensile strain along the same direction (Fig. 2b).

Remarkably, small levels of compressive strain along [1 1 0] barely affect the magnon energy, as illustrated in Fig. 2c. At larger strain values, $\left|{\varepsilon }_{[110]}\right| > 0.03\%$, the magnon energy increases notably, as in the case of [1 0 0] strain. Further increasing strain leads to rapid suppression of the peak intensity and significant broadening of the peak. Tensile strain along [1 1 0] induces a similar behavior, as shown in Fig. 2d. Overall, the magnon peak is extremely sensitive to orthorhombic deformation, as an external strain of 0.1% increases the magnon energy by almost 40%. This finding clearly demonstrates an intimate connection between the in-plane magnetic anisotropy and the lattice deformation.

**Fig. 3:**
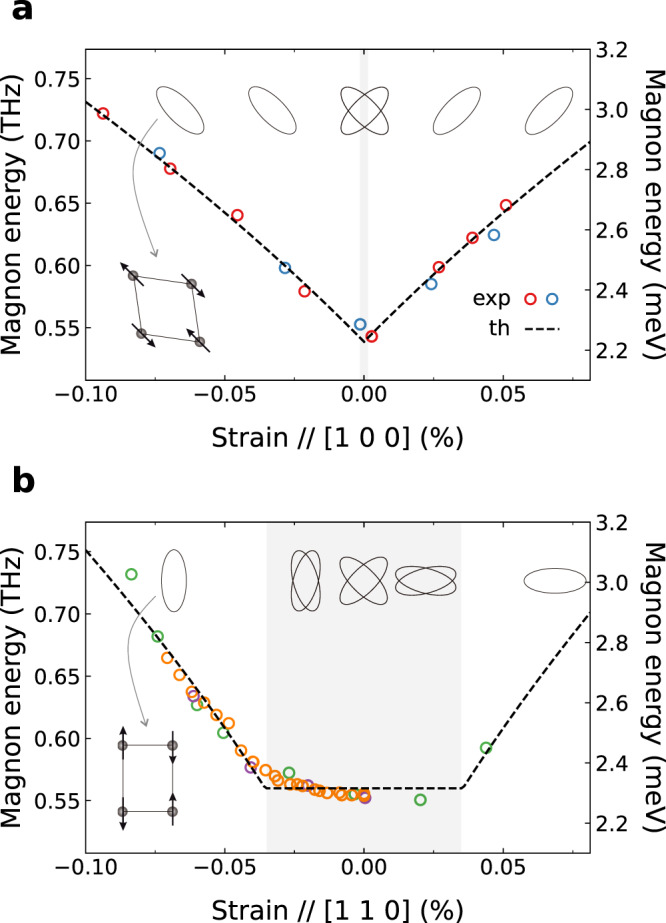
Response of magnon energies to uniaxial stress. Magnon energy as a function of uniaxial strain along the **a** [1 0 0] and **b** [1 1 0] axes. Circles with different colors represent data points measured from different samples. They are vertically shifted by the following magnitudes (in THz): red(0), blue(0.02), green(0), orange(0.02), and magenta(0.04). Different vertical shifts are likely due to slightly different sample qualities. Dashed lines are derived from the pseudospin-lattice coupling theory. Ellipses represent (exaggerated) orthorhombically-distorted magnetic domains, as illustrated on the lower left side of each figure. Two ellipses on top of each other correspond to two degenerate domains, coexisting in the shaded regions.

Figure 3 summarizes the dependence of the magnon energy on strain along different directions. Note that the magnon response is symmetric against the strain sign, i.e., the response to compressive and tensile strain is identical within the experimental error. This observation rules out any influence of interlayer interactions via expansion (contraction) of the lattice along the out-of-plane direction due to the Poisson ratio in response to in-plane compressive (tensile) strain^23^. In this scenario, the resulting change of the interlayer coupling and magnon energies should be monotonic functions of the out-of-plane lattice constant and hence antisymmetric with respect to the strain sign, in contrast to our observations.

We now show that the above symmetry, as well as the strong dependence of the magnon gap on the strain direction, are consistent with the pseudospin-lattice coupling picture. Although lattice distortions cannot lift the Kramers degeneracy of the $\widetilde{J}=1/2$ doublet, they modify the orbital content of the pseudospin wavefunctions via the admixture of higher-level states by Jahn-Teller coupling. As a result, new terms are generated in the exchange Hamiltonian. For uniform orthorhombic deformations *ε*_1_ = (*b* − *a*)/(*b* + *a*) and *ε*_2_ = (*y* − *x*)/(*y* + *x*) (depicted in Fig. 1c), they read^24^:

1 $${{{{{{{{\mathcal{H}}}}}}}}}_{s-l}={g}_{1}{\varepsilon }_{1}\,({S}_{i}^{x}{S}_{j}^{y}+{S}_{i}^{y}{S}_{j}^{x})+{g}_{2}{\varepsilon }_{2}\,({S}_{i}^{x}{S}_{j}^{x}-{S}_{i}^{y}{S}_{j}^{y}).$$

Hereafter, the pseudospin $\widetilde{J}$ is denoted by *S* = 1/2 for simplicity. One can interpret ${{{{{{{{\mathcal{H}}}}}}}}}_{s-l}$ as a coupling between the distortions (*ε*_1_, *ε*_2_) and pseudospin quadrupole moments (*Q*_1_, *Q*_2_), with ${Q}_{1}=({S}_{i}^{x}{S}_{j}^{y}+{S}_{i}^{y}{S}_{j}^{x})$ and ${Q}_{2}=({S}_{i}^{x}{S}_{j}^{x}-{S}_{i}^{y}{S}_{j}^{y})$ of *xy* and *x*^2^ − *y*^2^ symmetries, correspondingly. The quadrupoles *Q*_*γ*_ (*γ* ∈ 1, 2) reside on the nearest-neighbor exchange bonds 〈*ij*〉, and their amplitude is determined by short-range magnetic correlations. The constants *g*_*γ*_ depend on Jahn-Teller and SOC parameters^24^.

We define the staggered moment as $\overrightarrow{n}=S(\cos \alpha,\sin \alpha )$, with *α* = 0 corresponding to the Ir-O-Ir bond direction. This gives $({Q}_{1},{Q}_{2})=-{S}^{2}(\sin 2\alpha,\cos 2\alpha )$ per bond classically, and we obtain the pseudospin-lattice coupling energy (per site) in the following form:

2 $${E}_{s-l}=-2{S}^{2}\left({g}_{1}{\varepsilon }_{1}\sin 2\alpha+{g}_{2}{\varepsilon }_{2}\cos 2\alpha \right).$$

The linear dependence *E*_*s*−*l*_ ∝ *ε*_*γ*_ implies structural instability. Minimizing Eq. (2) together with the elastic energy of the lattice, $\frac{1}{2}{K}_{\gamma }{\varepsilon }_{\gamma }^{2}$, one finds the orthorhombic distortions

3 $${\varepsilon }_{1}=\frac{{\Gamma }_{1}}{{g}_{1}}\sin 2\alpha,\quad {\varepsilon }_{2}=\frac{{\Gamma }_{2}}{{g}_{2}}\cos 2\alpha,$$

where ${\Gamma }_{\gamma }=2{S}^{2}{g}_{\gamma }^{2}/{K}_{\gamma }$. The total energy as a function of the moment direction *α* then reads:

4 $$E=-{\Gamma }_{1}{S}^{2}+({\Gamma }_{1}-{\Gamma }_{2}){S}^{2}{\cos }^{2}2\alpha .$$

When Γ_1_ > Γ_2_, the anisotropy energy *E* is minimized at *α* = 45^∘^ or 135^∘^ (i.e., two types of domains), which corresponds to the case of Sr_2_IrO_4_^14,35^. The resulting distortion, which we denote as *ε*_0_ below, is of *xy* symmetry and equal to the *ε*_1_ value at *α* = 45^∘^, i.e., *ε*_0_ = Γ_1_/*g*_1_. This type of orthorhombic distortion is natural for perovskites, as it does not affect the metal-oxygen-metal bond length.

The magnon gap due to the intrinsic deformation *ε*_0_, caused by pseudospin-lattice coupling, is given by^24^:

5 $${\Delta }_{0}=8S\sqrt{J{\Gamma }_{1}}=8S\sqrt{J{g}_{1}{\varepsilon }_{0}},$$

which is about 0.51−0.58 THz^18,19,26^.

External strain *ε* affects the deformation pattern and thus the magnon energy. Strain applied along the [1 0 0] direction preserves the *xy*-type symmetry of the intrinsic distortion *ε*_0_ and hence simply enhances its amplitude (as soon as one of the two degenerate domains is selected by a very small external strain). As a result, the magnon gap increases monotonically as a function of *ε*:

6 $$\Delta (\varepsilon \parallel [1\,0\,0])=8S\sqrt{J{g}_{1}({\varepsilon }_{0}+|\varepsilon|)}={\Delta }_{0}\sqrt{1+\frac{|\varepsilon|}{{\varepsilon }_{0}}}.$$

This dependence is in excellent agreement with the experimental data (Fig. 3a). From corresponding fits, we obtain the magnon gap and intrinsic distortion values in Sr_2_IrO_4_:

7 $${\Delta }_{0}=0.54\,{{{{{{{\rm{THz}}}}}}}},\qquad {\varepsilon }_{0}=1.18\times 1{0}^{-3}.$$

Assuming *J* ~24 THz^15,36^, we evaluate *g*_1_ ~0.41 THz and Γ_1_ ~0.75 GHz.

**Fig. 4:**
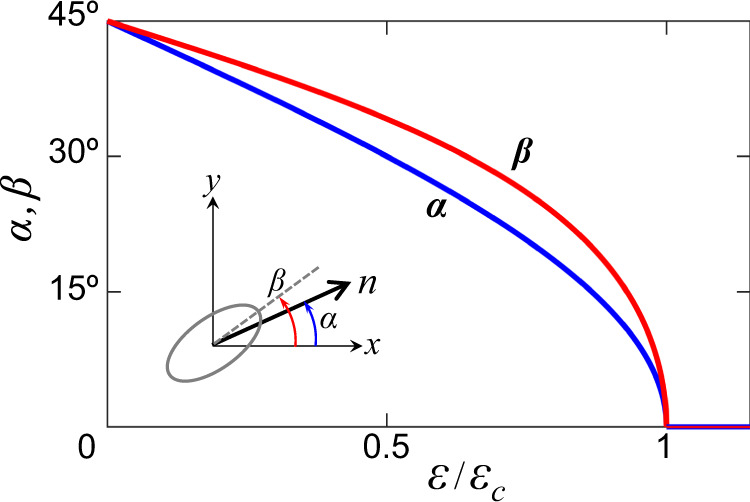
Rotation of the staggered moment and orthorhombic axis under uniaxial strain. Angles *α* and *β* which specify the staggered moment direction and lattice deformation axis (see inset), correspondingly, as a function of strain *ε* applied along the [1 1 0] direction.

The strain *ε* applied along the Ir-O-Ir bond direction [1 1 0] acts differently. It couples to the quadrupole *Q*_2_ of *x*^2^ − *y*^2^ symmetry, and thus contributes to the second term in Eq. (2): *ε*_2_ → (*ε*_2_ + *ε*). Minimizing the resulting *E*_*s*−*l*_ with respect to the distortions *ε*_*γ*_ and the angle *α*, we find two distinct regimes. At small values of strain, ∣*ε*∣ < *ε*_*c*_, where ${\varepsilon }_{c}={\varepsilon }_{0}\frac{{g}_{1}}{{g}_{2}}\left(1-\frac{{\Gamma }_{2}}{{\Gamma }_{1}}\right)$, the staggered moment and orthorhombic axis gradually rotate from the 45^∘^ diagonal ([1 0 0] axis) towards the Ir-O-Ir bond direction, as shown in Fig. 4. Specifically, we find $\cos 2\alpha=\varepsilon /{\varepsilon }_{c}$, with a slightly different angle *β* for the distortion axis given by $\tan 2\beta=\frac{{\varepsilon }_{1}}{{\varepsilon }_{2}+\varepsilon }=\frac{{g}_{2}}{{g}_{1}}\tan 2\alpha$. However, the magnetic anisotropy energy curvature is not affected by this rotation, and thus the magnon gap remains unchanged: Δ(*ε* < *ε*_*c*_) = Δ_0_.

For strain values ∣*ε*∣ > *ε*_*c*_, the lattice deformation axis and moment direction stay along the Ir-O-Ir bond direction (see Fig. 4), while the distortion amplitude and hence the magnon gap start to increase. We find

8 $$\Delta (\varepsilon > {\varepsilon }_{c})={\Delta }_{0}\sqrt{1+\frac{{g}_{2}}{{g}_{1}}\frac{|\varepsilon|-{\varepsilon }_{c}}{{\varepsilon }_{0}}}.$$

The theoretical results for *ε*∥[110] agree well with the data (Fig. 3b). We used the following fit parameters:

9 $$\begin{array}{rcl}{\Delta }_{0}=0.56\,{{{{{{{\rm{THz}}}}}}}},&&{\varepsilon }_{0}=1.18\times 1{0}^{-3},\\ {g}_{2}/{g}_{1}=1.43,&&{\varepsilon }_{c}=3.46\times 1{0}^{-4},\end{array}$$

where the value of *ε*_0_ is from Eq. (7). With the *ε*_*c*_/*ε*_0_ and *g*_2_/*g*_1_ values at hand, we evaluate the ratio Γ_2_/Γ_1_ = 0.58, in excellent agreement with its estimate (0.6^24^) obtained from the magnetization anisotropy data^26^. Notice that the magnon energy for ∣*ε*∣ < *ε*_*c*_ is not perfectly flat and shows rounding of the curve around *ε*_*c*_. This could be due to local defects and interfaces between domain boundaries affecting the local potential and, thus, the dynamics of domain rotation and subsequent elongation/compression. Alternatively, the presence of a small magnetic anisotropy could give rise to such rounding of the experimental curve, as discussed in Supplementary Note 2.

## Discussion

Overall, the pseudospin-lattice coupling theory accounts very well for our observations, especially the strong anisotropy of the strain effect. The latter is due to the presence of an intrinsic orthorhombicity, which is caused by the same pseudospin-lattice coupling that breaks the tetragonal symmetry of Sr_2_IrO_4_. From our data, we have quantified this distortion as *ε*_0_ ~10^−3^. Our results call for careful diffraction studies on high-quality single crystals of Sr_2_IrO_4_ to re-examine its crystal structure, which has so far been regarded as tetragonal. Interestingly, several reports on magnetoresistivity^37^ and neutron diffraction^38,39^ measurements also imply the presence of orthorhombic distortions in Sr_2_IrO_4_.

The observed giant response of the magnon energies to uniaxial stress is a direct fingerprint of unquenched orbital magnetism, which is naturally coupled to the crystal environment and provides an effective link between magnetism and lattice deformations. The theory of pseudospin-lattice interactions described here is generally applicable to other correlated-electron systems with spin–orbit-entangled magnetism. For example, it should form a basis for the understanding of magnetoelastic effects in the pseudospin $\widetilde{J}=1/2$ Kitaev model material RuCl_3_ and the $\widetilde{J}=0$ excitonic magnet Ca_2_RuO_4_.

We end our discussion with a few remarks on the potential of our results for the field of magnonics, which aims to develop high-speed electronic devices with minimal heating losses. The field has recently begun to focus on magnons with characteristic frequencies in the THz regime, which are common in antiferromagnets (including Sr_2_IrO_4_)^40–42^. However, manipulating the propagation of antiferromagnetic magnons on the requisite nanoscopic length scale presents a formidable challenge. Whereas the magnon energies are, in principle, susceptible to magnetic fields via the Zeeman interaction, it is difficult to engineer magnetic field configurations on this length scale. Analogous constraints apply to electric field manipulation of magnons in multiferroics. The uniaxial strain has been used to manipulate the ground states of complex antiferromagnets^43,44^, and some evidence has recently been reported for the sensitivity of ferromagnetic magnons at GHz frequencies to epitaxial^45^ or bending strain^46^. The extreme stress sensitivity of THz magnons we have discovered in a simple antiferromagnet opens up new opportunities for guiding magnons via strain domains in an otherwise homogeneous material, without resorting to heteroepitaxy and lithography^47^. Magnon conduits and magnonic crystals could then be fashioned from self-assembled strain domain patterns in thin-film structures, which can be readily reconfigured by force microscopy. Finally, the unexpectedly large stress response we have observed will greatly enhance the interaction of magnons and surface acoustic waves, which have been proposed as a platform for transient, reconfigurable magnonic crystals^42^. Whereas the feasibility of these design concepts remains to be explored, the results we have presented indicate that the giant stress response mediated by the spin–orbit interaction in 4*d*- and 5*d*-electron systems offer a powerful tuning parameter for the propagation of terahertz magnons.

## Methods

Since the magnetic structure of Sr_2_IrO_4_ is highly sensitive to chemical defects, high-quality Sr_2_IrO_4_ crystals were selected following the criteria discussed in the literature^34^. The crystals were cut into a needle shape using a wire saw and clamped into the strain device using the epoxy Stycast 2850FT. The applied stress was controlled by applying voltages on the piezoelectric stacks. The cutting process very often revealed a cleaved surface perpendicular to the c-axis, which is suitable for Raman experiments. All the data presented here were measured on cleaved surfaces. The beam spot of the laser (~10 × 10 μm^2^) on the sample is much smaller than the exposed area of the sample (~600 × 200 μm^2^), ensuring a homogeneous strain in the probing volume.

The Raman scattering experiments were performed in backscattering geometry using a JobinYvon LabRAM HR800 spectrometer from HORIBA. The 632.8 nm wavelength of a HeNe laser was chosen as the excitation line to maximize the magnetic scattering signals^25^. All the presented spectra under strain were measured at *T* = 20 K. In order to capture small energy shifts, a diffraction grating with 1800 grooves/mm was used. To avoid any measurement inconsistencies originating from spatial inhomogeneities, the Raman spectra were collected at the same position on the sample throughout the experiment. Due to the geometrical constraints of the experimental setup, the incident polarization was set to be parallel to the strain direction. As a result, depending on the strain direction, a different scattering configuration was required for magnon measurements in the *B*_2g_-symmetry: $c(ab)\bar{c}$ (*B*_2g_) for [1 0 0] strain and $c(xx)\bar{c}$ (*A*_1g_ + *B*_2g_) for [1 1 0] strain.

## Supplementary information

Supplementary information

## Data Availability

Data that support the findings of this study are available from the corresponding authors upon request.
