# Supplementary material for: Giant stress response of terahertz magnons in a spin-orbit Mott insulator
Source: Nat Commun. 2022 Nov 5;13:6674. doi: 10.1038/s41467-022-34375-6 (PMC9637169; doi:10.1038/s41467-022-34375-6)
Supplement: Supplementary file 1 — Supplementary information [file 41467_2022_34375_MOESM1_ESM.pdf]

Supplementary Information for
Giant stress response of terahertz magnons in a spin-orbit Mott insulator

Hun-Ho Kim,[1] Kentaro Ueda,[1,2] Suguru Nakata,[1] Peter Wochner,[1] Andrew Mackenzie,[3,4] Clifford Hicks,[3,5] Giniyat Khaliullin,[1] Huimei Liu,[1,6] Bernhard Keimer,[1] and Matteo Minola[1]

[1]*Max Planck Institute for Solid State Research, Heisenbergstraße 1, D-70569 Stuttgart, Germany*
[2]*Department of Applied Physics, The University of Tokyo, Bunkyo-ku, Tokyo, 113-8656, Japan*
[3]*Max Planck Institute for Chemical Physics of Solids, Nöthnitzer Straße 40, D-01187 Dresden, Germany*
[4]*SUPA, School of Physics and Astronomy, University of St. Andrews, St. Andrews KY16 9SS, United Kingdom*
[5]*School of Physics and Astronomy, University of Birmingham, Birmingham B15 2TT, United Kingdom*
[6]*Leibniz Institute for Solid State and Materials Research Dresden IFW, Helmholtzstraße 20, D-01069 Dresden, Germany*

Supplementary Note 1: Strain calibration using x-ray diffraction

Since experimental constraints imposed by the specific Raman setup used prevented us from x-ray diffraction measurements *in-situ*, the strain level was estimated during the Raman measurements using a built-in displacement sensor based on a parallel-plate capacitor, which has been proven to yield reasonable estimates in previous studies [1–6]. Nevertheless, subtle details such as the shape of the sample, the amount of epoxy used, and the epoxy thickness, can affect the accuracy of our strain estimation, thus making it difficult to precisely extract the parameters in our theory.

To obtain the best estimate of the strain present in the Raman experiments, we therefore performed separate x-ray diffraction measurements on the same sample mounted on the strain device to collect the Raman data. We controlled the sample strain by applying different voltages to the piezoelectric stacks of the strain device. This results in capacitance changes of the built-in displacement sensor, which is based on a parallel-plate capacitor. Using the inverse relation between the capacitance value and the displacement of a parallel-plate capacitor (C = $\varepsilon_0 A/d$, where C is the capacitance, ε_0 is the vacuum permittivity, A is the area of the parallel plates, and d is the distance between the plates), one obtains displacement changes for each strain step.

Figures S1(a),(c) show diffraction scans and Raman spectra as a function of displacement, respectively. From the x-ray measurements, we obtain a linear relation between the displacement and the lattice constant change, as shown in Figure S1(b). From the Raman scattering experiments, we extract a linear relation between the displacement and the energy of the phonon at 394 cm^{-1}, as displayed in Figure S1(d). Combining these two results, we obtain a phonon energy change against uniaxial strain of -0.188/0.0444 = -4.23 (cm^{-1}/%). This value, along with zero-strain references obtained from broken, fully relaxed samples after each strain experiment, was used to calibrate the strain scale of separate Raman experiments. The strain scale also includes the effect of the in-plane Poisson ratio of $\nu = 0.3$, which is the typical value for transition metal oxides with perovskite structure.

Notice that the first application of a small external stress applied along the [1 0 0] axis is only shifting twin domain boundaries without altering the intrinsic strain value ε_0, until a single domain type out of the two degenerate ones is selected and occupies the entire sample volume. Only after that, the local orthorhombic distortion starts to increase as $\varepsilon_0 + \varepsilon$, and one observes the magnon energy enhancement, as shown in Fig. 3(a) of the main text.

Supplementary Note 2: Interplay between spin-lattice coupling and spin-exchange anisotropy

In addition to the spin-lattice coupling mechanism, the in-plane magnon gap can also have contributions from the anisotropic spin exchange interactions. To describe this contribution on a phenomenological level, we introduce the following magnetic anisotropy energy E_{ex} of four-fold tetragonal symmetry:

$$E_{ex} = -\frac{1}{2}\kappa S^2 \cos 4\alpha. \tag{1}$$

A positive κ favors the easy axis along the Ir-Ir bond direction [110] (i.e. $\alpha = 0$), while $\kappa < 0$ favors spin direction along the [100] diagonal ($\alpha = \pi/4$). Microscopically, the bond-directional pseudodipolar exchange interaction in Sr_2IrO_4 generates exactly this type of anisotropy, with $\kappa > 0$, via a quantum order-by-disorder mechanism. Having a quantum nature, this term should be weak compared to the spin-lattice mechanism that operates on a classical level, via a lattice symmetry-breaking phase transition. For generality, we consider both signs of κ.

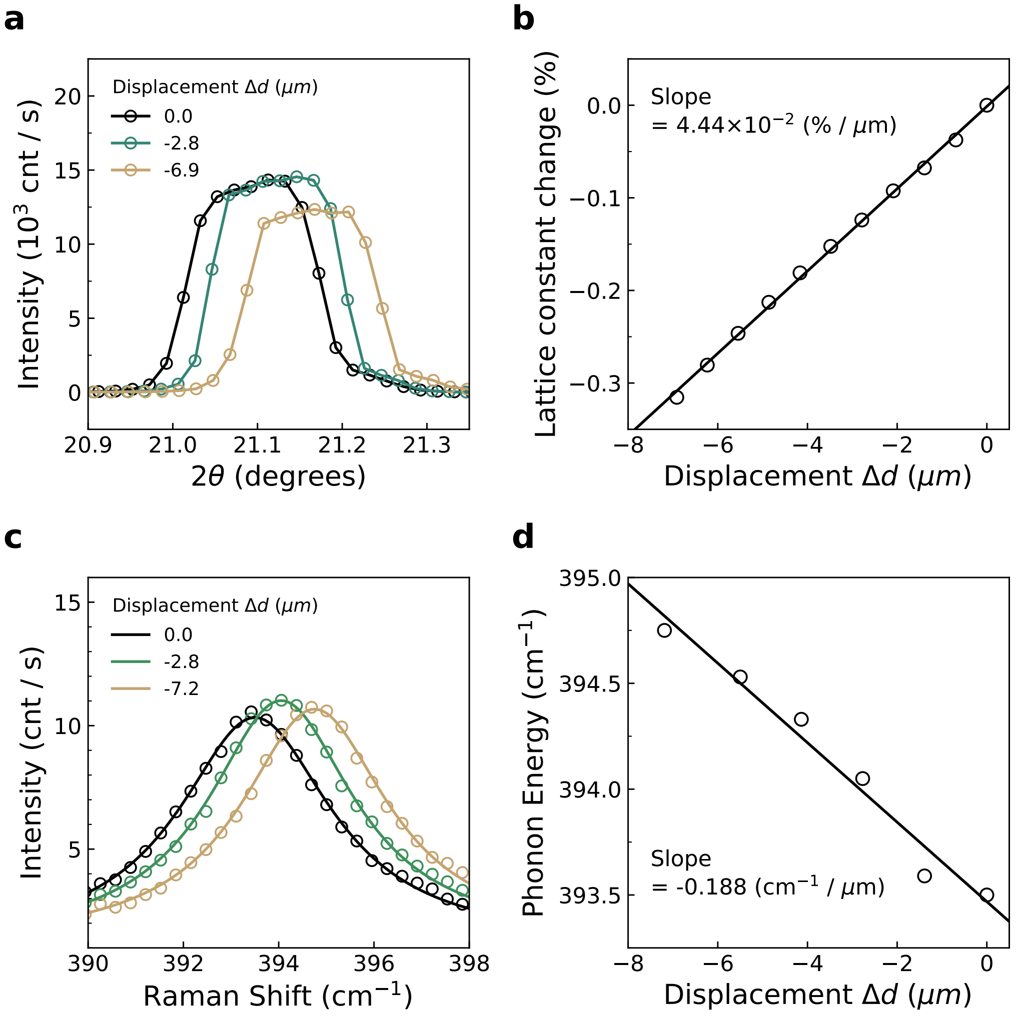

FIG. S1. **Comparison between x-ray and Raman data under uniaxial strain.** **a**, 2θ-scan of the (2 2 0) Bragg reflection measured at different displacement values. The solid lines are guides-to-the-eye. **b**, Lattice constant changes based on the shift of the (2 2 0) Bragg reflection as a function of displacement. The solid line is the result of a linear fit to the data. **c**, Raman spectra of the phonon at 394cm^{-1} measured at different displacement values. The solid lines are the results of Lorentzian fits to the data. **d**, Energy of the phonon at 394 cm^{-1} as a function of displacement. The solid line is the result of a linear fit to the data.

The total anisotropy energy $E(\alpha) = E_{s-l} + E_{ex}$, including spin-lattice as well spin-exchange anisotropy, reads as:

$$E(\alpha) = const + \frac{1}{2}(\Gamma_1 - \Gamma_2 - \kappa)S^2 \cos 4\alpha. \tag{2}$$

In order to have the moments along the diagonal direction ($\alpha = \pi/4$) as in Sr_2IrO_4, the parameter κ must obey the constraint $\kappa < \Gamma_1 - \Gamma_2$.

The magnon gap at zero strain $\varepsilon = 0$ is thus determined by the combined effect of the spin-lattice and spin-exchange anisotropies:

$$\Delta(\varepsilon = 0) = 8S\sqrt{J(\Gamma_1 - \kappa)} = \Delta_0\sqrt{1 - \frac{\kappa}{\Gamma_1}}. \tag{3}$$

Here, $\Delta_0 = 8S\sqrt{J\Gamma_1}$ is a magnon gap induced by spin-lattice contribution alone. Note that the pseudodipolar exchange anisotropy with $\kappa > 0$ reduces the gap value, as it favors spins along the Ir-Ir bond direction, thereby competing with the spin-lattice coupling (which dictates easy axis along [100]).

When the strain is applied along [1 0 0] direction, the κ term does not affect a functional form of the gap, *i.e.* the magnon gap monotonically increases as a function of the external strain ε:

$$\Delta(\varepsilon) = \Delta_0\sqrt{1 - \frac{\kappa}{\Gamma_1} + \frac{|\varepsilon|}{\varepsilon_0}} = \Delta(\varepsilon = 0)\sqrt{1 + \frac{|\varepsilon|}{\widetilde{\varepsilon}_0}}\,. \tag{4}$$

Here $\widetilde{\varepsilon}_0 = \varepsilon_0(1 - \frac{\kappa}{\Gamma_1})$, and $\varepsilon_0 = 2S^2 g_1/K_1$ is the spin-lattice induced spontaneuos orthorhombic distortion. The external strain [100] does not change the easy axis direction but monotonically increases the orthorhombicity and the size of the magnon gap.

External strain applied along [1 1 0] direction rotates the orthorhombicity axis and spin direction towards the Ir-Ir bond direction $\alpha = 0$, which is reached at some value of $\varepsilon = \varepsilon_c$ (see Fig. 4 of the main text). In the spin-lattice coupling theory, this rotation does not affect the magnon gap, as it is only sensitive to the amplitude of the distortion but not to its direction. Above ε_c, the easy axis remains along the [110] direction, but the orthorhombic distortion and hence the magnon gap start to increase. The κ term originating from spin-exchange anisotropy results in a new feature. Namely, the magnon gap becomes a function of strain even below ε_c:

$$\Delta(\varepsilon \le \varepsilon_c) = \Delta_0\sqrt{1 - \frac{\kappa}{\Gamma_1}\left(1 - \frac{\varepsilon^2}{\varepsilon_c^2}\right)}. \tag{5}$$

Here, a critical strain value $\varepsilon_c = \varepsilon_0\frac{g_1}{g_2}\left(1 - \frac{\Gamma_2}{\Gamma_1} - \frac{\kappa}{\Gamma_1}\right)$ is renormalized by κ. We note that at $\varepsilon = \varepsilon_c$ the magnon gap equals the spin-lattice contribution Δ_0.

For the strain values $\varepsilon > \varepsilon_c$, we obtain a monotonical increase of the gap as in a pure spin-lattice coupling theory (cf. Eq. 8 of the main text):

$$\Delta(\varepsilon > \varepsilon_c) = \Delta_0\sqrt{1 + \frac{g_2}{g_1}\frac{|\varepsilon| - \varepsilon_c}{\varepsilon_0}}\,. \tag{6}$$

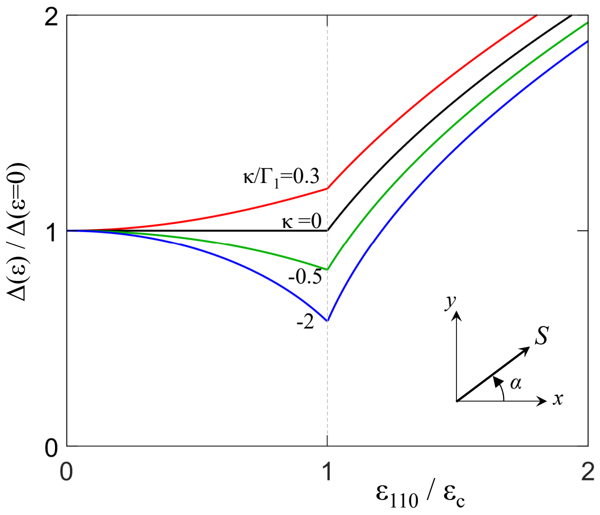

FIG. S2. Magnon gap as a function of strain along 110 direction at different κ/Γ_1 values.

The above equations determine the magnon gaps as a function of the external strain ε; the results for different κ values are shown in Fig. S2. The regime change in the magnon gap behavior at ε_c is preserved, but the kink at this point varies with κ. We see that the negative κ dependences are qualitatively different from the observed behavior and thus can be excluded. This is expected as the pseudodipolar exchange interaction results in $\kappa > 0$.

To quantify the value of κ in Sr_2IrO_4, we use the above equations to fit the experimental data. The results for $\kappa/\Gamma_1 = 0.03$, shown in Fig. S3, well reproduce the data, including a variation of the gap at small strain values $\varepsilon_{110} < \varepsilon_c$. We see that while the spin-exchange anisotropy term κ may improve a detailed comparison with experiment,

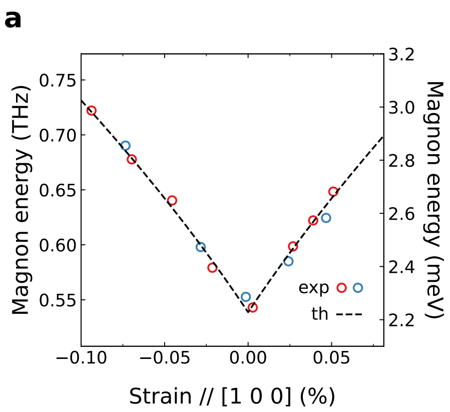

FIG. S3. Fitting the experimental data including the spin-exchange anisotropy κ term. The parameters used are $\kappa/\Gamma_1 = 0.03$, $\varepsilon_0 = 1.1 \times 10^{-3}$, $\varepsilon_c = 3 \times 10^{-4}$, and $g_2/g_1 = 1.1$.

it is actually very weak in Sr_2IrO_4 (just about a 0.03 fraction of the spin-lattice coupling energy Γ_1). This is not surprising because the exchange Hamiltonian in Sr_2IrO_4 is dominated by the isotropic Heisenberg coupling. Moreover, the anisotropic exchange terms like pseudodipolar coupling contribute to the in-plane magnetic anisotropy only via quantum (order-by-disorder) effects, further reducing their impact on the magnon gaps. We thus conclude that the spin-lattice coupling is the major source of the magnetic anisotropy in Sr_2IrO_4, as we considered in the main text.

[1] Hicks, C. W., Barber, M. E., Edkins, S. D., Brodsky, D. O. & Mackenzie, A. P. Piezoelectric-based apparatus for strain tuning. *Rev. Sci. Instrum.* **85**, 065003 (2014).

[2] Hicks, C. W. *et al.* Strong Increase of T_c of Sr_2RuO_4 Under Both Tensile and Compressive Strain. Science **344**, 283 (2014).

[3] Steppke, A. *et al.* Strong peak in T_c of Sr_2RuO_4 under uniaxial pressure. *Science* **355**, 9398 (2017).

[4] Barber, M. E., Gibbs, A. S., Maeno, Y., Mackenzie, A. P. & Hicks, C. W. Resistivity in the Vicinity of a van Hove Singularity: Sr_2RuO_4 under Uniaxial Pressure. *Phys. Rev. Lett.* **120**, 076602 (2018).

[5] Kim, H.-H. *et al.* Uniaxial pressure control of competing orders in a high-temperature superconductor. *Science* **362**, 1040 (2018).

[6] Kim, H.-H. *et al.* Charge Density Waves in $YBa_2Cu_3O_{6.67}$ Probed by Resonant X-Ray Scattering under Uniaxial Compression. *Phys. Rev. Lett.* **126**, 037002 (2021).
